# Putative hydrophobins of black poplar mushroom (*Agrocybe cylindracea*)

**DOI:** 10.1080/21501203.2020.1804474

**Published:** 2020-08-31

**Authors:** Chetsada Pothiratana, Wasapon Fuangsawat, Anchalee Jintapattanakit, Churapa Teerapatsakul, Surachai Thachepan

**Affiliations:** a Department of Microbiology, Faculty of Science, Kasetsart University, Bangkok, Thailand; b Center for Advanced Studies in Tropical Natural Resources, NRU-KU, Kasetsart University, Bangkok, Thailand; c Department of Pharmacy, Faculty of Pharmacy, Mahidol University, Bangkok, Thailand; d Department of Chemistry, Faculty of Science, Kasetsart University, Bangkok, Thailand; e Kamnoetvidya Science Academy, Rayong, Thailand

**Keywords:** Hydrophobin, water contact angle, emulsifier, surface tension, LC-MS/MS

## Abstract

Hydrophobin proteins were extracted from *Agrocybe cylindracea* mycelia, the culture media (potato dextrose broth, PDB), and fruiting bodies. The putative hydrophobins obtained showed approximate sizes ranging from 8.0 to 25.0 kDa, dependent on their source. Multiple hydrophobin protein bands were detected in fruiting bodies. The hydrophobin yielded from aerial mycelia, or fruiting bodies, was approximately 6 mg/g dried weight. The crude extracts were examined for their properties in regards to surface modification, emulsification, and surface activity. Coating of hydrophobic Teflon sheet with crude extract made the surface significantly hydrophilic, whereas exposure of glass surfaces to extracts resulted in enhanced hydrophobicity. Crude extracts from culture media of *A. cylindracea* displayed emulsifying activity when mixed with hexane and could significantly reduce the surface tension of 60% ethanol and deionised water. The putative hydrophobin protein band from culture media (9.6 kDa), as analysed using LC-MS/MS, contained an amino acid fragment structurally similar to class I hydrophobin proteins from Basidiomycetes.

## Introduction

Hydrophobins are a group of small proteins (typically < 20 kDa) found only in filamentous fungi and mushrooms. A key feature of each of these proteins is the presence of eight conserved cysteine (Cys) residues, which form four specific disulphide bonds. Although few similarities in amino acid sequence are evident in this group, very similar hydropathy patterns are observed. While hydrophobins are considered moderately to highly hydrophobic, these molecules are amphipathic which promotes their self-assembly into monolayers at hydrophilic:hydrophobic interfaces (Sunde et al. 2008; Bayry et al. 2012; Ren et al. 2013). According to their biophysical properties and hydropathy patterns hydrophobins can be divided into two classes (I and II). Class I hydrophobins form rodlets that are highly insoluble and resistant to dissociation even at elevated temperature (2% SDS at 100°C), although this can be achieved on treatment with formic or trifluoroacetic acids. In contrast, class II hydrophobins can dissolve in 60% ethanol or 2% SDS (Wösten and de Vocht 2000).

Class I hydrophobins are more widely distributed than class II hydrophobins, for the latter is restricted to Ascomycetes (Linder et al. 2005). Hydrophobins are essential to the life cycle of these fungi, stimulating the emergence and formation of aerial hyphae or fruiting bodies and promoting the attachment of fungal cells on hydrophobic surfaces (Wösten and de Vocht 2000; Linder et al. 2005; Ren et al. 2013). Hydrophobin genes are usually found as multigene families in several fungi. Most fungal species contain 2–7 hydrophobin genes with the exception of the mushroom *Coprinus cinereus*, which has up to 23 genes (Sunde et al. 2008; Ren et al. 2013). This latter observation has paved the way for their application in the medical and pharmaceutical fields (e.g. for protein immobilisation), and as food emulsifiers (Scholtmeijer et al. 2001; Linder et al. 2005; Tchuenbou-Magaia et al. 2009; Zampieri et al. 2010). Additionally, the SC3 hydrophobin from *Schizophyllum commune* has been shown to exhibit antitumor activity (Akanbi et al. 2013).

*Agrocybe cylindracea*, known as the black poplar mushroom, is an edible mushroom widely cultured in East Asia due to its taste and texture (Uhart and Albertó 2007; Alam et al. 2010). While the hypoglycaemic, mitogenic and immunomodulating activities of the polysaccharides and lectins in *A. cylindracea* have been documented (Kiho et al. 1994; Yoshida et al. 1996; Wang et al. 2002), the properties of hydrophobins from this species and their potential applications have not been explored. Accordingly, we were interested in investigating hydrophobin proteins at different growth stages of *A. cylindracea*, and studying their properties as prelude to further biotechnological and medical applications.

In this study, putative hydrophobins from *Agrocybe cylindracea* were extracted from mycelia, culture media, and fruiting bodies. These were characterised and compared in terms of their size, emulsifying property, surface activity, and ability to modify both hydrophobic and hydrophilic surfaces.

## Materials and methods

### Mushroom

Black poplar mushroom (*A. cylindracea*) was purchased from the Department of Agriculture (Bangkok, Thailand) and maintained on Potato Dextrose Agar (PDA) at room temperature (30 ± 2°C). Its identification was confirmed using PCR-based techniques as previously described (Teerapatsakul et al. 2017) (using universal primers ITS1 and ITS4).

### Chemicals and media

All chemicals used were of analytical reagent grade. Potato Dextrose Broth (PDB) and PDA were purchased from TM Media, India, and Criterion, USA, respectively.

### Cultivation of A. cylindracea

Aerial mycelia of *A. cylindracea* were grown in static liquid cultures. An inoculum was prepared from a mycelial colony cultivated on PDA at room temperature. Three agar plugs (5 mm × 5 mm) taken from the edge of the colony were inoculated into a 500 mL Erlenmeyer flask containing 150 mL of PDB. The culture was cultivated stationarily at room temperature (30 ± 2°C) for 20–30 days until mycelia covered the entire surface. Mycelia and culture media were then separated by vacuum filtration, then the mycelia were stored at −80°C and the culture media were stored at 4°C prior to use.

Fruiting bodies of *A. cylindracea* were obtained through cultivation of the mushrooms in sawdust, which were contained in plastic bags. For cultivation of fruiting bodies, mycelia of *A. cylindracea* on PDA were added into a bottle of autoclaved-sterile sorghum seeds, and the bottle was incubated at room temperature for 2 weeks. The mycelia growing on sorghum seeds were then transferred into autoclaved-sterile sawdust bags, and the bags were then incubated at 28–30°C. At the point of full mycelia growth, the lids were removed to induce the formation of fruiting bodies. The fruiting bodies were harvested after 3 days.

### Hydrophobin extraction and protein electrophoresis analysis

Hydrophobins were extracted using previously described methods (Lugones et al. 1996; Tchuenbou-Magaia et al. 2009). Mushroom mycelia and fruiting bodies were first ground in liquid nitrogen, then treated with hot 1% SDS buffer for 10 min, and then washed 4–6 times with deionised water. For static liquid culture media, it was blended using an electric blender to obtain a stable foam, prior to freeze-drying. After freeze-drying, the hydrophobins were extracted from the sample by treatment with trifluoroacetic acid (TFA) overnight at 0°C. After evaporation of the TFA using a stream of nitrogen gas, the remaining pellet was dissolved in 60% (v/v) ethanol and allowed to stand overnight at room temperature. Undissolved material was then removed by filtration (0.22 μm filter), and the crude hydrophobin extract was stored at 4°C.

The hydrophobin concentration in the crude extract was determined using the Bradford Assay (Bradford 1976). The crude hydrophobins were separated using 16% Tricine-Sodium Dodecyl Sulphate-Polyacrylamide gel electrophoresis (Tricine-SDS-PAGE) (Schägger and Von Jagow 1987), with extract samples being loaded at a concentration of 3 µg/lane. Polyacrylamide gels were stained with Coomassie Brilliant Blue R250 for 20 min, and then destained until the protein bands were clearly visible (Bollag et al. 1996).

### Water contact angle (WCA) measurements

WCA measurements of mushroom colony surfaces cultivated on PDA were done via a modification of a published protocol (Chau et al. 2009), while those of hydrophobin-coated surfaces were measured as previously described (Vigueras et al. 2014). These involved images of 0.1% aniline blue droplets (2 µL) on each surface being captured using a digital camera and the WCA values being calculated using Image_J software using the Contact_Angle plug-in.

### Coating of hydrophilic and hydrophobic surfaces

The technique used for coating surfaces was a modification of a previously published procedure (Vigueras et al. 2014). Glass slides and Teflon (PTFE) sheets were used as reference materials for hydrophilic and hydrophobic surfaces, respectively. All sheets were washed 3 times with ethanol and then distilled water prior to being coated with 100 μL of crude hydrophobin extract (150 µg/mL) over a 0.5 cm^2^ area. After air-drying at room temperature for 48 h, the sheets were boiled in 1% hot SDS for 10 min to remove unbound non-hydrophobin proteins, and then washed with distilled water. After further air-drying (48 h), WCA measurements were conducted. Surface coating with ethanol 60% (v/v) was used as negative control in all cases.

### Determination of surface tension

Crude hydrophobin extract (50 μg/mL) in either 60% (v/v) ethanol or deionised water were used for surface tension determination using a DropMaster DM-CE1 instrument (Kyowa Interface Science Co., Ltd, Japan). Images of droplets hanging on a 20 G-SUS syringe with 8 μL 60% (v/v) ethanol and 13 μL deionised water extract solutions, respectively, were captured 10 times in 1000 ms at 26°C using the ds/dC automatic mode. FAMAS software was used to calculate surface tension from these images.

### Emulsifying property

To study the emulsifying property of extracts, 1.5 mL of hexane was vigorously mixed with an equal volume of crude hydrophobin extract (approximately 120 μg/mL) in 60% ethanol in a 12 × 75 mL test tube. After capping the tube and standing for 24 h, the extent of emulsion after 24 h (%E24) was calculated in accordance with the following equation (Masakorala et al. 2013).

$$\%E24=\left(\frac{Height\;\;of\;\;emulsion\;\;layer(cm)}{Height\;\;of\;\;whole\;\;solution(cm)}\right)\times 100$$

### Identification of proteins in extracts using LC-MS/MS

Hydrophobins were separated from the crude extract using Tricine-SDS-PAGE, with protein band being excised after Coomassie Brilliant Blue R250 staining. Each protein sample was then washed, reduced, alkylated, and trypsinised (digestion), prior to amino acid sequence analysis using LC-MS/MS (LC: Dianex ultimate 3000/column: hypersil GOLD size 50 × 0.5 mm, MS: MS: ESI-trap-Amazon SL BRUKER).

### Statistical analysis

Statistical analysis in this study utilised one-way ANOVA Duncan variance, assuming a 95% confidence interval, and was undertaken using the SPSS statistic V.23 IBM software package.

## Results and discussion

### Characterisation and identification of A. cylindracea

**Figure 1.**
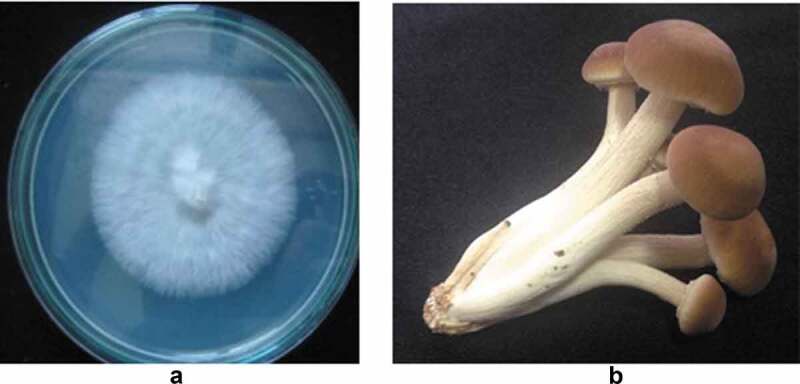
Characteristics of *Agrocybe cylindracea*. (a) Morphology of a 7-d colony grown on PDA (b) 3-d fruiting bodies harvested from sawdust

The identity of the *A. cylindracea* strain used in this study was confirmed by blasting a sequence of the internal transcribed spacer (ITS) region of the nuclear ribosomal DNA, amplified by universal primers ITS1 and ITS4, against the Genbank database on the NCBI website. The ITS sequence of this mushroom strain showed 100% sequence similarity to *Agrocybe cylindracea* strain NAAS03938 (accession no: KP004974), with an E-value of 0%. The morphology of *A. cylindracea* colonies grown on PDA is shown in Figure 1(a). Examination of the water contact angle (WCA) value of a 10-d old colony surface (134.7 ± 1.2°) showed the level of hydrophobicity to be in line with results by Chau et al. (2009), who reported that filamentous fungi such as *Alternaria* sp., *Penicillium aurantiogriseum* and *Cladosporium* spp. grown on PDA also exhibit similar WCA values (122° to 145°) (Chau et al. 2009). Fruiting bodies of this mushroom strain, as shown in Figure 1(b), show pilei being brown with white margins and stripes, which is consistent with the literature description of *A. cylindracea* (Uhart and Albertó 2007).

### Protein electrophoresis of crude hydrophobin extracts from aerial mycelia, culture media, and fruiting bodies of A. cylindracea

Hydrophobin proteins were extracted from aerial mycelia, culture media, and fruiting bodies of *A. cylindracea*, following a standard protocol as previously described (Lugones et al. 1996; Tchuenbou-Magaia et al. 2009), and were then dissolved in 60% ethanol. Protein concentrations and yields of hydrophobins extracted from mycelia, culture media and fruiting bodies of *A. cylindracea* are summarised in Table 1.

**Table 1.** Concentration and yield of hydrophobin extracts from aerial mycelia, culture media, and fruiting bodies of *Agrocybe cylindracea.*

Source	Protein concentration (μg/mL)	Yield (a = mg/g dry weight, b = mg/L)
Aerial mycelia	73.2–321.4	1.46–6.42^a^
Culture media	38.4–127.5	1.42–1.96^b^
Fruiting bodies	307.9–376.7	6.15–6.16^a^

**Figure 2.**
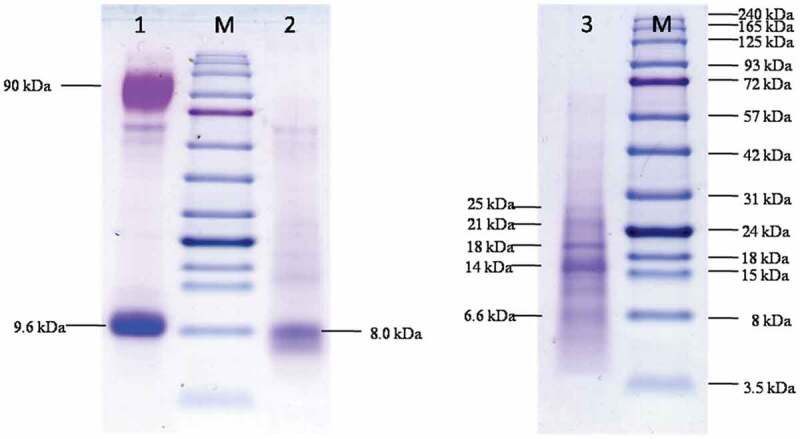
Protein Electrophoresis of hydrophobin extracts from aerial mycelia, culture media and fruiting bodies of *Agrocybe cylindracea* using 16% Tricine-SDS PAGE loading with 3 mg protein per lane

The highest yield of hydrophobins was obtained from aerial mycelia (6.42 mg/g dry weight), with the majority being class I hydrophobins bound to the cell wall (4.9 mg/g biomass) (Vigueras et al. 2008). PDB culture media contained secreted hydrophobins at levels approximating 1.42–1.96 mg/L. Fruiting bodies of *A. cylindracea* yielded 6.16 mg of hydrophobin/g dry biomass weight. Crude hydrophobin extracts from mycelia and culture media were analysed using Tricine-SDS-PAGE. Extracts in PDB culture media contain a hydrophobin band (approximate size 9.6 kDa) in addition to a further 90 kDa protein band (Figure 2 lane 1) which may arise from other secreted proteins such as hydrolytic enzymes. Crude extracts from mycelia afforded a major, single, dominant band corresponding to hydrophobin (approximate size of 8.0 kDa), which is suggestive of high protein purity (Figure 2 lane 2). Crude hydrophobin extracts obtained from fruiting bodies consist of five protein bands having sizes of 6.6, 14, 18, 21 and 25 kDa. (Figure 2 lane 3). Unlike vegetative mycelia, mushroom fruiting bodies utilise several hydrophobins, each of which exhibits a specific function such as coating the outer surface of these systems, or lining air channels within them. In *Agaricus bisporus*, the ABH3 hydrophobin is coated on hyphal surfaces, whereas ABH1 is particularly abundant on the outer surface of fruiting bodies, resulting in their hydrophobic nature (De Groot et al. 1996; Lugones et al. 1996). Additionally, *Schizophyllum commune* has hyphal surfaces coated with SC3 hydrophobin, with SC4 being found lining air channels within fruiting bodies, which may prevent the collapse of air channels within them and support gas exchange under wet conditions. In *Schizophyllum commune* SC4 are found in four isoforms, having sizes of 8.32, 8.39, 8.4, and 8.7 kDa (Lugones et al. 1999).

Lane M = marker, lane 1 = crude hydrophobin extract from culture media, lane 2 = crude hydrophobin extract from aerial mycelia, lane 3 = crude hydrophobin extract from fruiting bodies.

### Surface modification properties

The ability of crude hydrophobin extracts from aerial mycelia, culture media, and fruiting bodies to modify the properties of both hydrophilic and hydrophobic surfaces was investigated. Glass slides and Teflon sheet were employed as representatives of respective hydrophilic and hydrophobic surfaces. Coating of glass slides with crude hydrophobin extracts obtained from culture media and fruiting bodies resulted in significant increases in water contact angle, from 54.3° ± 13.1 (glass) to 78.5° ± 8.1 and 65.2° ± 2.8, respectively, whereas those from extracts of aerial mycelia showed no significant differences (Table 2). These findings are consistent with those utilising hydrophilic mica surfaces, which become hydrophobic when coated with EAS hydrophobin (Ren et al. 2013).

**Table 2.** Water Contact Angle (WCA) values of glass slides and Teflon sheets coated with crude hydrophobin extracts (150 μg/mL) from aerial mycelia, culture media, and fruiting bodies of *Agrocybe cylindracea.*

	Glass slide		Teflon sheet	
Sample coating	WCA (^o^) (Mean ± SD)	Image	WCA (^o^) (Mean ± SD)	Image
Control (60% ethanol)	54.3 ± 13.1^a^		107.9 ± 6.1^a^	
Aerial mycelia	59.8 ± 7.8^ab^		79.1 ± 10.5^b^	
Culture media	78.5 ± 8.1^b^		66.0 ± 16.8 ^c^	
Fruiting bodies	65.2 ± 2.8^b^		59.3 ± 17.6 ^c^	

Mean values in the same column followed by different superscripts are significantly different. (*p* < 0.05), *n* = 13–15

In contrast, coating Teflon sheets with crude hydrophobin extracts resulted in the surfaces becoming significantly more hydrophilic. As shown in Table 2, coating of Teflon with crude hydrophobin extracts from mycelia, culture media, and fruiting bodies resulted in significant decreases in water contact angle from 107.9° ± 6.1 (Teflon) to 79.1° ± 10.5, 66.0° ± 16.8 and 59.3° ± 17.6, respectively. In this study, all crude hydrophobin extracts from different sources exhibited surface modification properties as typically found in hydrophobins, albeit to different extents. Past work on Teflon surface coating using SC3 and SC4 hydrophobins (20 μg/mL) from *Schizophyllum commune*, with hot surfactant treatment resulted in decreases in WCA values from 115° to 55° and 68°, respectively (Scholtmeijer et al. 2002), while coating with ABH1, the hydrophobin of *Agaricus bisporus*, at a concentration of 20 μg/mL with hot 1% SDS treatment also resulted in significant enhancements in surface hydrophilicity (Lugones et al. 1996).

### Determination of surface tension

**Figure 3.**
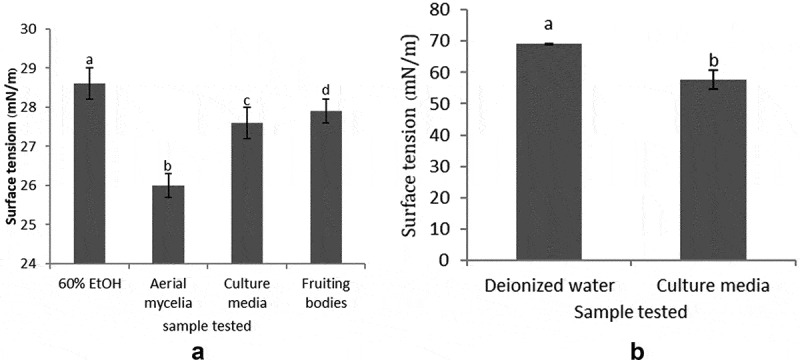
Surface tension of hydrophobin extracts (50 μg/mL) dissolved in (a) 60% ethanol and (b) deionised water. Graphs represent mean values and error bars represent SD values. Mean values indicated with the same lowercase letter are not significantly different. (*n* = 10)

As highlighted in Figure 3, crude hydrophobin extracts from mycelia, culture media, and fruiting bodies were able to alter the surface tension of solutions relative to the control. For hydrophobin extracts in 60% ethanol from mycelia, culture media and fruiting bodies, this equated to reductions in surface tension from 28.6 ± 0.4 mN/m (control) to 26.0 ± 0.3, 27.6 ± 0.4 and 27.9 ± 0.3 mN/m, respectively (Figure 3(a)). For comparison, the total protein from crude hydrophobin extract of culture media was precipitated with acetone and re-dissolved in deionised water, prior to measuring surface tension. At a concentration of 50 μg/mL, the hydrophobins significantly reduce surface tension, from 69.2 ± 0.1 mN/m in deionised water to 57.6 ± 3.0 mN/m (Figure 3(b)). Therefore, crude hydrophobin extracts of *A. cylindracea* exhibit surfactant properties. In filamentous fungi, hydrophobins are secreted into culture media to reduce water surface tension, prior to hyphae escaping the aqueous phase to form aerial mycelia (Wösten et al. 1999). In a similar way, the hydrophobin PLHYD from *Paecilomyces lilacinus* at a concentration of 1.47 mg/mL was able to reduce surface tension of water from 70 to 34.8 mN/m (Vigueras et al. 2014), and 100 μg/mL HFBI, HFBII, and SC3 also reduce surface tension values of water from 72 to 42, 35 and 27 mN/m, respectively (Askolin et al. 2006).

### Emulsifying properties

**Figure 4.**
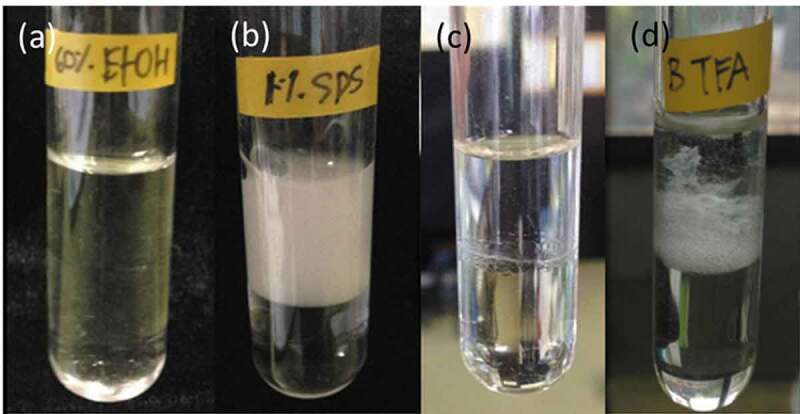
Appearance of emulsion layer from a mixture of hexane and (a) 60% ethanol, (b) 1% SDS, (c) crude hydrophobin extracts from aerial mycelia, and (d) from culture media, after 24 h at room temperature

Due to their amphipathic nature, crude hydrophobin extracts were examined for their ability to act as emulsifiers. Crude hydrophobin extracts from aerial mycelia and culture media at concentrations of approximately 120 μg/mL showed %E24 values of 6.6% and 25%, respectively. Solutions of 1% SDS and 60% ethanol were used as positive and negative controls, giving %E24 values of 50% and 0%, respectively. The emulsion layer obtained from the aerial mycelia extract contained small air bubbles whereas that from culture media was white with the presence of small cotton-like aggregates. Emulsions from SDS solution were white without any aggregated material being present (Figure 4(a–d)). The difference in %E24 values and appearance of emulsion layers were possibly due to their different emulsifying properties, which is suggestive of their different roles during the mushroom life cycle. It is possible that secreted hydrophobins promote the solubilisation of the hydrophobic substrate into the aqueous phase, resulting in the formation of a stable emulsion which increases the bioavailability of the substrate to the fungi. However, the presence of the high molecular weight protein in crude hydrophobin extract from culture media may also play roles in dictating its emulsifying and surface tension properties.

### Identification of proteins in extracts using LC-MS/MS

**Figure 5.**
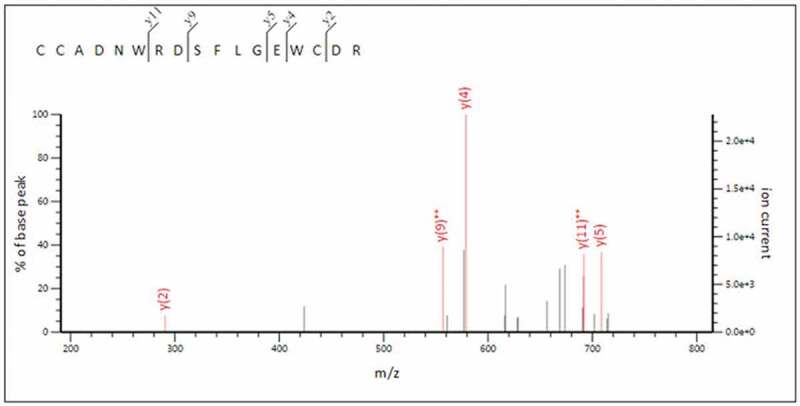
Peptide Sequence analysis of peptide fragment no. 16 using LC-MS/MS

**Figure 6.**
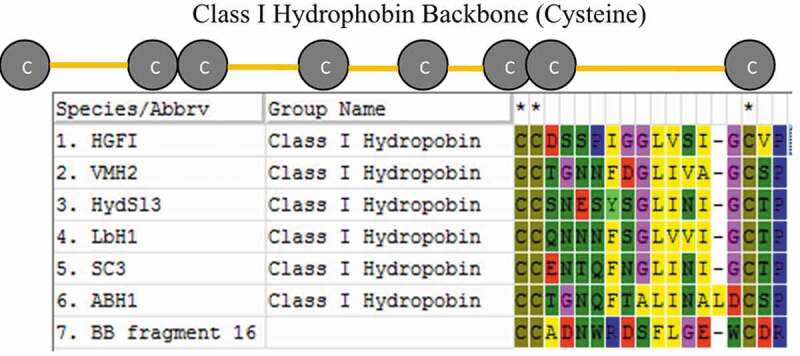
Alignment of peptide fragment no. 16 (BB fragment 16) with respective region between conserved cysteine 6–8 of other hydrophobins: HGFI; *Grifola frondosa* (accession no. ABO42329), VMH2; *Pleurotus ostreatus* PC15 (accession no. KDQ22759), Hyd3; *Suillus luteus* UH-Slu-LM8-n1 (accession no. KIK42959), LBH1; *Laccaria bicolour* S238 N-H82 (accession no. EDR13197), SC3; *Schizophyllum commune* (accession no. P16933) and ABH1; *Agaricus bisporus* (accession no. P49072)

Each protein band from hydrophobin extracts was purified using SDS-PAGE and subjected to trypsin digestion, prior to sequence analysis using LC-MS/MS. Amino acid sequences obtained were searched for sequence similarity on the Mascot protein database (www.matrixscience.com). From these results, no sequence similarity was found to entries in the database, which may arise from low sequence similarities among hydrophobin protein members (Sunde et al. 2008) or the *A. cylindracea* annotated genome sequence being unavailable. We then turned our attention towards the characteristic cysteine residues in hydrophobin proteins. The 9.6 kDa protein band extracted from culture media contained one 17 amino acid fragment, peptide no. 16, out of 37 peptide fragments, sequence CCADNWRDSFLGEWCDR (Figure 5). This protein contains three cysteine residues in a pattern similar to those observed for conserved cysteine residues in class I hydrophobins, with the two adjacent cysteine residues being probably conserved cysteine no. 2–3 or no. 6–7. When aligned with other class I hydrophobin sequences from mushrooms, peptide no. 16 showed similar length and amino acid patterning relative to hydrophobin sequences for the region between cysteine no. 6 to 8 (Figure 6), which suggests that the peptide no. 16 fragment originates from a class I hydrophobin protein in *A. cylindracea*. This amino acid sequence can be used to design degenerate primers in order to amplify this gene in *A. cylindracea*.

* indicate conserved cysteine; C: cysteine, Hydrophobic amino acid; F: phenylalanine, L: leucine, I: isoleucine, A: alanine, V: valineHydrophilic amino acid; S: serine, T: threonine, N: asparagine, Q: glutamine, W: tryptophan.

## Conclusions

Putative hydrophobins in black poplar mushroom (*Agrocybe cylindracea*) were extracted from its aerial mycelia, culture media, and fruiting bodies. Major single protein bands in putative hydrophobins were found in aerial mycelia and culture media whereas multiple bands were found in fruiting bodies. Hydrophobin yields of approximately 6 mg/g dried weight were obtained from aerial mycelia and fruiting bodies, which were relatively high compared to the average value. All crude hydrophobin extracts in this study featured characteristics of previously reported hydrophobins such as their ability to act as emulsifiers, having surface activity, and the ability to modify the hydrophobic/hydrophilic property of material surfaces, which make them attractive for biotechnological and medical applications. However, further studies in purification of hydrophobins are required for these applications to be fully realised. Accordingly, *A. cylindracea*, an important commercially cultivated edible mushroom with customer acceptance, can be viewed as a viable candidate for hydrophobin production.
